# The Knowledge-Practice Gap in Primordial Hypertension Prevention Through Social Determinants of Health Among Normotensive Adults in Rural South Africa

**DOI:** 10.3390/healthcare14010011

**Published:** 2025-12-20

**Authors:** Monwabisi Faleni, Laston Gonah, Guillermo Alfredo Pulido Estrada, Sibusiso Cyprian Nomatshila

**Affiliations:** 1 School of Public Health, Faculty of Medicine and Health Sciences, Walter Sisulu University, Mthatha 5100, South Africa; lgonah@wsu.ac.za (L.G.); gpulidoestrada@wsu.ac.za (G.A.P.E.); snomatshila@wsu.ac.za (S.C.N.); 2 WSU Society and Health Research Institute, Walter Sisulu University, Mthatha 5100, South Africa

**Keywords:** hypertension, primordial prevention, knowledge, attitudes, practices, social determinants of health, South Africa, rural communities

## Abstract

**Background:** Hypertension is a leading modifiable risk factor for cardiovascular diseases globally, disproportionately affecting low- and middle-income countries (LMICs), including South Africa. Primordial prevention targeting normotensive individuals plays a key role in reducing lifetime risk. **Aim:** To assess knowledge, attitudes and practices (KAP) and social determinants of health related to primordial hypertension prevention among normotensive adults in OR Tambo District, Eastern Cape province. **Methods:** A community-based cross-sectional study was conducted among 245 randomly selected normotensive adults. A validated questionnaire captured socio-demographic characteristics and KAP levels. Data analysis included descriptive statistics, Cronbach’s alpha, and chi-square tests (*p* < 0.05). **Results:** Participants demonstrated moderate knowledge (53.9%), highly positive attitudes (86.1%), and fair preventive practices (59.6%), highlighting a clear knowledge–practice gap. Higher knowledge was significantly associated with female gender (*p* < 0.001), older age (*p* < 0.001), and family history of hypertension (*p* = 0.001). Positive attitudes correlated with older age (*p* = 0.018) and higher education (*p* = 0.008). Knowledge level significantly predicted both positive attitudes (*p* < 0.001) and preventive practices (*p* = 0.009). **Conclusions:** Despite moderate knowledge and positive attitudes, a clear knowledge–practice gap was evident, possibly influenced by social and structural constraints. Strengthening primordial hypertension prevention in rural South Africa requires integrated strategies combining context-specific health education with interventions addressing structural barriers to enable sustainable behaviour change.

## 1. Introduction

Hypertension remains a global public health priority, representing a major risk factor for cardiovascular diseases (CVD), stroke, and renal failure [1]. Globally, approximately 1.28 billion adults live with hypertension, with nearly half unaware of their condition, highlighting persistent gaps in awareness, detection, and early preventive engagement [2]. Low- and middle-income countries (LMICs), including South Africa, bear a disproportionate burden due to structural and systemic barriers such as inadequate healthcare infrastructure, limited preventive services, and low health literacy, resulting in suboptimal detection and control rates [3]. Urbanization, dietary transitions toward energy-dense foods, physical inactivity, and psychosocial stressors further exacerbate hypertension prevalence, imposing both social and economic strain on vulnerable populations [4].

In South Africa, hypertension is a pervasive health issue, with adult prevalence estimates ranging from 40% to 50% [5]. Despite this high burden, awareness, treatment, and control rates remain suboptimal: only about half of affected adults are reportedly aware of their condition, and even fewer achieve adequate blood pressure control [6,7,8]. These challenges are particularly pronounced in resource-constrained provinces such as the Eastern Cape, where limited healthcare access, poverty, and structural inequities hinder effective management, even among diagnosed individuals [7,9].

Primordial prevention, which aims to prevent the emergence of risk factors before they develop, is increasingly recognized as a cornerstone strategy for addressing hypertension [10,11]. By targeting normotensive individuals, this approach promotes the early adoption of healthy behaviours and the creation of supportive environments to reduce lifetime cardiovascular risk [12]. Within this context, the Knowledge, Attitudes, and Practices (KAP) framework provides a useful lens for understanding how awareness, beliefs, and risk perception shape preventive behaviours and inform the design of effective interventions [13]. Evidence shows that community-level programmes promoting healthy diet or dietary modification, physical activity, and stress management play an important role in fostering a culture of prevention [14].

However, the effectiveness of KAP-driven prevention efforts is profoundly shaped by social determinants of health (SDH). Factors such as education, income, employment, gender, language, infrastructure, and food security can either be facilitators or barriers to the adoption of preventive practices or behaviours [15,16]. In rural South African settings, structural inequities; including poverty, low health literacy, and limited access to nutritious foods and safe recreational facilities, may impede the translation of knowledge and positive attitudes into sustained preventive behaviours [15,17,18,19,20]. Broader system-level factors, including health-system capacity, policy environments, and intersectoral coordination, can further influence opportunities for prevention [1,18]. This underscores that individual knowledge and motivation alone may be insufficient to achieve lasting behavioural change.

Against this background, this study sought to assess KAP related to primordial hypertension prevention among normotensive adults in OR Tambo District, Eastern Cape. By examining how KAP intersect with key social determinants of health, the study provides evidence to inform context-specific, multi-level strategies that address both individual and structural barriers, supporting more effective and sustainable hypertension prevention in rural populations.

## 2. Methods

### 2.1. Study Design and Setting

A community-based cross-sectional study was conducted between May and August 2025 in OR Tambo District, a predominantly rural and underserved region of the Eastern Cape Province, South Africa. The district is characterized by limited healthcare infrastructure, high unemployment, and substantial socioeconomic disparities, which may influence health behaviours and access to preventive services.

### 2.2. Study Population and Sampling

The study population comprised normotensive adults aged 18 years and older, confirmed by systolic blood pressure (SBP) < 140 mm of mercury (mmHg) and diastolic blood pressure (DBP) < 90 mmHg. Individuals with a prior diagnosis of hypertension or those currently on antihypertensive medication were excluded. A sample size of 245 participants was determined using Cochran’s formula [21] with a 95% confidence level and a 6% margin of error.

A multistage cluster sampling strategy was employed to select the sample of 245 normotensive adults from the OR Tambo District. In the first stage, all five local municipalities, namely, King Sabata Dalindyebo, Mhlontlo, Nyandeni, Port St Johns, and Ingquza Hill, were included. Two primary healthcare facilities were then randomly selected from each municipality, yielding ten sites representing both rural and peri-urban settings. In the final stage, participants were recruited through systematic random sampling from outpatient departments, with every kth eligible individual approached until the allocated quota for each facility was reached. Facility-specific quotas were determined proportionate to the facility catchment population to maintain balanced representation.

### 2.3. Data Collection Tool and Procedure

Data were collected using a structured questionnaire, adapted from validated KAP instruments [22,23,24,25] to ensure content validity, and pre-tested in a pilot study of 20 participants from a comparable community. The questionnaire was revised based on pretest findings to enhance clarity, cultural relevance, and comprehension. The questionnaire was administered by trained fieldworkers in the participants’ preferred language (isiXhosa or English) and captured socio-demographic characteristics (age, gender, education, employment, marital status, distance to healthcare facilities, and family history of hypertension), knowledge of hypertension (causes, risk factors, complications), attitudes toward prevention (perceived importance and feasibility of healthy behaviours), and preventive practices (dietary habits, physical activity, salt intake, alcohol/tobacco use). Internal consistency of each KAP domain was assessed using Cronbach’s alpha with α ≥ 0.80 indicating good reliability, 0.70–0.79 acceptable, 0.6–0.69 borderline; and <0.60 considered low reliability. Domain scores were categorized using sample-based percentiles as low (≤33 rd), moderate (34th–66th), or high (≥67th), enabling standardized comparisons across participants.

### 2.4. Data Analysis

Data were analysed using SPSS version 28.0. Descriptive statistics (frequencies and percentages) were used to summarise socio-demographic characteristics and KAP levels. Associations between socio-demographic variables and KAP categories were tested using chi-square or Fisher’s exact tests where assumptions were not met. A *p*-value < 0.05 was considered statistically significant.

### 2.5. Ethical Considerations

Ethical approval was obtained from the Walter Sisulu University Human Research Ethics Committee (WSU HREC 016/2525). Written informed consent was obtained from all participants prior to data collection, and confidentiality and anonymity were strictly maintained throughout the study.

## 3. Results

### 3.1. Socio-Demographic Characteristics

A total of 245 normotensive adults participated in the study. Most participants were female (61.6%), and the age distribution showed a predominance of middle-aged adults (45–54 years, 41.2%), followed by younger adults (18–24 and 25–34 years, each 18.4%), adults aged 35–44 years (13.9%), and older adults (55+ years, 8.2%). Education levels were predominantly secondary (60.8%), followed by tertiary (21.6%) and primary (13.9%). Most participants were single (68.2%), unemployed (69.4%), and just over half (51.8%) reported a family history of hypertension. The majority resided within 5–15 km of the nearest health facility, although 21.2% lived more than 15 km away (Table 1).

### 3.2. Knowledge, Attitude, Practice, and Belief Levels

Regarding hypertension-related KAP, participants demonstrated moderate knowledge (53.9%), highly positive attitudes (86.1%), and fair preventive practices (59.6%), illustrating a knowledge–practice gap (Table 2). Cronbach’s alpha indicated good reliability for Knowledge (α = 0.81) and Attitudes (α = 0.82) sections, and borderline reliability for Lifestyle/Preventive Practices (α = 0.62) and Beliefs (α = 0.60) which is considered minimally acceptable for exploratory community-based studies, supporting the questionnaire’s overall suitability.

### 3.3. Associations Between KAP Domains and Sociodemographic Characteristics

Significant associations were observed between KAP domains and key sociodemographic characteristics. Knowledge levels varied by gender, age, and family history of hypertension, with women, older adults, and those reporting a family history of hypertension more likely to demonstrate moderate or high knowledge (*p* < 0.001, *p* < 0.001, and *p* = 0.001, respectively), as shown in Table 3. Attitude levels were similarly influenced by age (*p* = 0.018) and education (*p* = 0.008), with older and more educated participants expressing more positive attitudes toward primordial hypertension prevention (Table 4). Preventive practice levels were not significantly associated with gender, age, education, or employment status.

Cross-domain relationships demonstrated strong internal consistency between KAP variables. Knowledge was significantly associated with attitudes (*p* < 0.001), with higher knowledge corresponding to more positive attitudes (Table 5). A similar pattern was observed between knowledge and preventive practices (*p* = 0.009), where participants with higher knowledge more frequently reported fair or good practice levels (Table 6).

Collectively, these results highlight a knowledge-practice gap mediated by social and structural determinants.

## 4. Discussion

This study provides critical insights into primordial hypertension prevention among normotensive adults in a rural South African context. The findings reveal a population with moderate knowledge, highly positive attitudes, but only moderate engagement in preventive practices, highlighting a clear and persistent knowledge–practice gap.

The observed moderate knowledge level aligns with evidence from other LMICs, where awareness of hypertension risk factors often exists without deep understanding of their implications and associated complications [2,26]. Notably, knowledge was significantly higher among women, older adults, and individuals with a family history of hypertension, suggesting that both gendered health engagement patterns and personal/familial risk perception influence learning. Interestingly, formal education was not significantly associated with knowledge, indicating that in rural South African settings, health information may be acquired through community interactions, lived experience, or informal channels rather than formal schooling, a phenomenon documented in other resource-constrained regions [27].

The overwhelmingly positive attitudes reflect a receptive environment for public health interventions. Positive attitudes were significantly associated with older age and higher education, reinforcing the importance of cognitive engagement and experience in shaping health beliefs [28,29]. However, the moderate level of preventive practices confirmed that knowledge and attitudes alone do not guarantee behaviour change. This attitude-behaviour disconnect is well-documented, particularly in contexts where structural constraints limit the translation of awareness into action [30].

Social determinants of health (SDH) are key mediators in this gap. High unemployment, poverty, geographic barriers to healthcare, limited access to nutritious foods, and inadequate spaces for physical activity act as structural impediments that constrain individuals’ capacity to adopt or sustain healthy lifestyles, despite their knowledge and positive attitudes [20,31]. Even participants with high knowledge predominantly engaged in moderate preventive practices, proving that information alone is insufficient in the absence of supportive environments [18]. Macro-level determinants, including health system capacity and policy frameworks, shape the adoption of healthy practices yet involve multiple sectors, highlighting the need for multi-level interventions to sustain behaviour change.

### 4.1. Implications for Policy and Practice

The findings highlight that hypertension prevention in rural South Africa must move beyond traditional health education toward multi-level, context-responsive interventions. Strengthening community structures such as peer networks, walking groups, and local wellness initiatives can embed preventive behaviours into daily routines. A Health in All Policies (HiAP) approach, promoting multi-sectoral collaboration across agriculture, food and trade regulation, education, and local governance, is essential to address the broader social determinants that shape health choices. Programmes should also leverage women, children and families with a history of hypertension as pivotal community change agents, supported by culturally grounded behaviour-change communication that translates positive attitudes into sustained preventive practices. Involving children provides early exposure to healthy behaviours, establishes lifelong habits, and reinforces preventive practices across generations through culturally grounded behaviour-change communication. Finally, integrating community-based screening and counselling within routine primary care can reinforce early prevention and continuity of care, advancing equitable cardiovascular health outcomes.

### 4.2. Limitations

The study’s cross-sectional design limits causal inference. Despite validation and pre-testing for clarity and cultural relevance, self-reported attitudes and practices may be affected by reporting or social desirability bias, especially among normotensive participants with limited prior hypertension exposure. Findings may not generalize beyond similar rural, underserved communities, and the lower reliability observed in the practices section underscores the need for improved measurement tools in future research. Additionally, the analysis relied on cross-tabulations and chi-square tests, which do not account for confounding or provide predictive insight. Future studies with larger samples should consider multivariable or ordinal logistic regression to better explore determinants of hypertension-related knowledge, attitudes, and practices.

### 4.3. Recommendations

Based on study findings on knowledge, attitudes, practices, and social determinants, future interventions should:

- Use local behavioural data to design information, education, and communication (IEC) programmes that are context-specific, actionable, and culturally relevant.
- Co-create interventions with communities to ensure ownership, alignment with local norms, and improved adoption of preventive behaviours.
- Regulate the food and trade environment, including production, marketing, and importation of ultra-processed and high-sodium foods, to reduce exposure to obesogenic products and promote healthier diets.
- Adopt a Health in All Policies approach, fostering cross-sector collaboration (agriculture, trade, education, local governance) to tackle upstream determinants such as poverty, food insecurity, and limited access to safe recreational spaces.
- Empower women and family units as central agents of behavioural change, leveraging their influence within households and communities to reinforce preventive practices.
- Strengthen primary healthcare engagement through routine community-based screening, counselling, and follow-up to facilitate early detection and sustain long-term preventive behaviours.

## 5. Conclusions

Normotensive participants in OR Tambo District demonstrated foundational knowledge and positive attitudes toward hypertension prevention, but a substantial gap between knowledge and practice persists, driven by socioeconomic and structural constraints. Effective primordial hypertension prevention urgently requires integrated strategies combining tailored health promotion and literacy programs, community-based participatory interventions, and multi-sectoral actions on social determinants of health via a Health in All Policies approach. Addressing these upstream barriers is critical for translating knowledge into sustained preventive behaviours and reducing the future burden of hypertension.

## Figures and Tables

**Table 1:** Socio-Demographic and Health-Related Characteristics of Participants (N = 245).

Variable	n (%)
Gender	
▪ Male	94 (38.4)
▪ Female	151 (61.6)
Age	
▪ 18–24	45 (18.4)
▪ 25–34	45 (18.4)
▪ 35–44	34 (13.9)
▪ 45–54	101 (41.2)
▪ 55+	20 (8.2)
Education	
▪ None	9 (3.7)
▪ Primary	34 (13.9)
▪ Secondary	149 (60.8)
▪ Tertiary	53 (21.6)
Employment	
▪ Employed/Self-employed	75 (30.6)
▪ Unemployed	170 (69.4)
Distance to the nearest facility	
▪ <5 km	55 (22.4)
▪ 5–9 km	80 (32.7)
▪ 10–15 km	58 (23.7)
▪ >15 km	52 (21.2)
Family History of Hypertension	
▪ Yes	127 (51.8)
▪ No	118 (48.2)

**Table 2:** Knowledge, Attitude, Practice, and Belief Levels (N = 245).

Variable	n (%)
Knowledge Level	
▪ Low	42 (17.1)
▪ Moderate	132 (53.9)
▪ High	71 (29.0)
Attitude Level	
▪ Negative	9 (3.7)
▪ Neutral	25 (10.2)
▪ Positive	211 (86.1)
Practice Level	
▪ Poor	17 (6.9)
▪ Fair	146 (59.6)
▪ Good	82 (33.5)
Belief Level	
▪ Low	9 (3.7)
▪ Moderate	19 (7.8)
▪ High	217 (88.6)

**Table 3:** Association between socio-demographic characteristics and Knowledge levels among participants (N = 245).

Variable	Low n (%)	Moderate n (%)	High n (%)	*p*-Value
Gender				<0.001 *
▪ Male	23 (24.5)	58 (61.7)	13 (13.8)	
▪ Female	19 (12.6)	74 (49.0)	58 (38.4)	
Age Group				<0.001 *
▪ 18–24	14 (31.1)	19 (42.2)	12 (26.7)	
▪ 25–34	6 (13.3)	20 (44.4)	19 (42.2)	
▪ 35–44	10 (29.4)	11 (32.4)	13 (38.2)	
▪ 45–54	8 (7.9)	79 (78.2)	14 (13.9)	
▪ 55+	4 (20.0)	3 (15.0)	13 (65.0)	
Education				0.390
▪ None	3 (33.3)	3 (33.3)	3 (33.3)	
▪ Primary	7 (20.6)	17 (50.0)	10 (29.4)	
▪ Secondary	27 (18.1)	83 (55.7)	39 (26.2)	
▪ Tertiary	5 (9.4)	29 (54.7)	19 (35.8)	
Employment				0.770
▪ Employed	12 (16.0)	43 (57.3)	20 (26.7)	
▪ Unemployed	30 (17.6)	89 (52.4)	51 (30.0)	
Family history HPT				0.001 *
▪ Yes	11 (8.7)	79 (62.2)	37 (29.1)	
▪ No	31 (26.3)	53 (44.9)	34 (28.8)	

* Bold values indicate statistically significant associations (*p* < 0.05).

**Table 4:** Association between socio-demographic characteristics and Attitude levels among participants (N = 245).

Variable	Negative n (%)	Neutral n (%)	Positive n (%)	*p*-Value
Gender				0.523
▪ Male	4 (4.3)	12 (12.8)	78 (82.9)	
▪ Female	5 (3.3)	13 (8.6)	133 (88.1)	
Age Group				0.018 *
▪ 18–24	3 (6.7)	7 (15.6)	35 (77.8)	
▪ 25–34	2 (4.4)	6 (13.3)	37 (82.2)	
▪ 35–44	1 (2.9)	3 (8.8)	30 (88.2)	
▪ 45–54	3 (3.0)	3 (3.0)	95 (94.0)	
▪ 55+	0 (0.0)	6 (30.0)	14 (70.0)	
Education				0.008 *
▪ None	0 (0.0)	4 (80.0)	5 (20.0)	
▪ Primary	0 (0.0)	6 (21.4)	28 (78.6)	
▪ Secondary	8 (6.0)	9 (6.8)	132 (87.2)	
▪ Tertiary	1 (1.9)	6 (11.3)	46 (86.8)	
Employment				0.903
▪ Employed	2 (2.7)	8 (10.7)	65 (86.7)	
▪ Unemployed	7 (4.1)	17 (10.0)	146 (85.9)	

* Bold values indicate statistically significant associations (*p* < 0.05).

**Table 5:** Association between Knowledge Levels and Attitude Levels among Participants (N = 245).

Knowledge Level	Negative Attitude n (%)	Neutral Attitude n (%)	Positive Attitude n (%)	Total	*p*-Value
▪ Low	8 (19.0)	15 (35.7)	19 (45.2)	42	**<0.001** *
▪ Moderate	1 (0.8)	5 (3.8)	126 (95.5)	132	
▪ High	0 (0.0)	5 (7.0)	66 (93.0)	71	
Total	9 (3.7)	25 (10.2)	211 (86.1)	245	

* Bold values indicate statistically significant associations (*p* < 0.05).

**Table 6:** Association between Knowledge Levels and Practice Levels among Participants (N = 245).

Knowledge Level	Poor Practices n (%)	Fair n (%)	Good Practices n (%)	Total	*p*-Value
▪ Low	6 (14.3)	29 (69.0)	7 (16.7)	42	**0.009** *
▪ Moderate	9 (6.8)	69 (52.3)	54 (40.9)	132	
▪ High	2 (2.8)	48 (67.6)	21 (29.6)	71	
Total	17 (6.9)	146 (59.6)	82 (33.5)	245	

* Bold values indicate statistically significant associations (*p* < 0.05).

## Data Availability

The data presented in this study are available from the corresponding author on reasonable request due to ethical restrictions imposed by the authorizing ministry.
